# Overweight patients benefit from high tibial osteotomy to the same extent as patients with normal weights but show inferior mid-term results

**DOI:** 10.1007/s00167-021-06457-3

**Published:** 2021-02-11

**Authors:** Moritz Herbst, Marc-Daniel Ahrend, Leonard Grünwald, Cornelius Fischer, Steffen Schröter, Christoph Ihle

**Affiliations:** 1 Department of Traumatology and Reconstructive Surgery, BG Trauma Center Tübingen, Eberhard Karls University Tübingen, Schnarrrenbergstr. 95, 72076 Tübingen, Germany; 2 AO Research Institute Davos, Davos, Switzerland; 3 Department of Traumatology and Reconstructive Surgery, Diakonie Klinikum GmbH Jung-Stilling-Krankenhaus, Siegen, Germany

**Keywords:** High tibial osteotomy, Open-wedge HTO, Quality of life, Obesity

## Abstract

**Purpose:**

The purpose of this prospective study was to analyze the impact of obesity on the clinical and radiological outcomes 6 years after open-wedge high tibial osteotomy (HTO).

**Methods:**

A total of 120 prospectively recorded patients with medial compartment osteoarthritis underwent open-wedge HTO between 2008 and 2011. The study cohort was frequently examined over a minimum of a 6-year follow-up. The cohort was divided into three groups according to body mass index (BMI): normal weight patients (BMI < 25 kg/m^2^), pre-obese patients (BMI 25–30 kg/m^2^) and obese patients (BMI > 30 kg/m^2^). Clinical and functional outcomes (Oxford Knee Score, Hospital for Special Surgery Score, Lequesne Score, Tegner Activity Scale), subjective health-related quality of life (SF-36), change in mechanical limb alignment (mTFA) as well as conversion to unicompartmental or total knee arthroplasty (TKA) were evaluated. To compare clinical scoring between the groups, univariate variance analysis was applied. Changes in outcome variables over time were analyzed with dependent *t* tests.

**Results:**

From 120 patients, 85 were followed-up over a 6.7-year period on average (6–11.8 years) after HTO. The mean BMI was 28.6 ± 4.6 kg/m^2^. Each group showed a significant pre- to postoperative increase in all recorded scores *(p* < *0.05).* In absolute terms, both mental and clinical scores of overweight patients did not reach the peak values of the normal weighted population during the period of observation. There was a conversion to TKA in 10.5% after an average of 50.1 ± 25.0 months following surgery. A total of five complications occurred without significant differences (BMI < 25: *n* = 1, BMI 25–30: *n* = 2, BMI > 30: *n* = 2; *n.s.).* There was a mean pre- to postoperative (six weeks after surgery) correction difference of 6.9° ± 3.2° (mTFA) with higher loss of correction over time in overweight patients.

**Conclusion:**

In terms of clinical outcome and health-related quality of life, overweight patients may receive a benefit from open-wedge HTO to the same extent as patients with normal weights and show similar complication rates. However, they have inferior preoperative clinical and functional results and mid-term results after open-wedge HTO compared to patients with normal weights.

**Level of evidence:**

Level III.

## Introduction

Medial open-wedge HTO using an angular locking plate system as described by Staubli et al. [35] as well as Lobenhoffer and Agneskirchner [23] has been reported to achieve good to excellent mid- to long-term results with low complication rates [4, 35].

Several studies have investigated the potential risk factors that lead to inferior subjective and functional outcomes after HTO surgery. Female sex [12, 37], mental illness, impaired psychological situation, and previous surgeries were found to be risk factors for poorer surgical outcome [20, 27, 31, 37]. The impact of an older age on surgical outcomes [7, 10, 21, 37] remains controversial. Additionally, low preoperative values for the subjective health-related quality of life (HRQL) are associated with poor postoperative functional results [13].

Obesity is known as a major risk factor for complications and impaired clinical outcome following different types of knee surgery [1, 15]. Registry-based data have indicated higher complication rates in overweight patients after total knee arthroplasty and particularly in frequent periprosthetic joint infections [1, 36]. Studies on unicompartmental knee arthroplasty have found a fivefold increase in failure rate in morbidly obese patients due to disease progression in other compartments or mobile bearing instability [26].

Open-wedge HTO is most frequently recommended for young and active patients with unicompartmental varus gonarthritis [7, 24]; however, data regarding the association between body weight and functional outcomes following this procedure remain limited. Studies investigating short-term outcomes have revealed inferior clinical and functional results for overweight patients [2, 6, 34]. However, no detailed analysis regarding mid-term outcome following HTO in overweight patients has been done. This analysis is essential given that obesity leads to a faster progression of knee osteoarthritis [3, 5].

The purpose of this study was to analyze the impact of obesity on mid-term outcomes following open-wedge HTO surgery. Therefore, the following outcome measures were used: conversion to knee arthroplasty (TKA), general perioperative complications, and change in mechanical limb alignment as well as clinical and functional outcomes. Special attention was paid to subjective HRQL, which is used to describe the success of treatment after surgery for degenerative joint disease [13, 16, 27]. The main hypothesis was that a high body mass index (BMI) leads to poor functional outcome.

## Materials and methods

Between 2008 and 2011, 120 consecutive and prospectively assessed patients underwent medial open-wedge HTO surgery. Inclusion criteria included symptomatic varus malalignment and medial compartment osteoarthritis or articular cartilage lesion of the knee. All patients underwent clinical and radiological examinations preoperatively and at three postoperative visits (6th month, 12th month, 18th month). A fourth follow-up was done at least 6 years after the open-wedge HTO. Exclusion criteria included patients’ rejection of study participation and missing contact information, as well as a change in BMI of more than 15% during the study period. Patients with traumatic, focal osteochondral lesions were also excluded from clinical and radiological evaluation. The study was approved by the local ethics committee (488/2014BO2).

The body weight and height of the patients were measured to calculate the preoperative BMI. Based on the calculated BMI, the cohort was divided into subgroups according to the World Health Organization international classification of adults’ body weight index [22]. The first group (group 1) was composed of all patients with normal weight (BMI < 25 kg/m^2^). The second group was composed of all pre-obese patients with a BMI between 25 and 30 kg/m^2^ (group 2), and the third group was composed of obese patients with a BMI > 30 kg/m^2^ (group 3).

### Clinical outcome

Body height, body weight, BMI, complications, and conversion to TKA were recorded at each follow-up. Additionally, the following established clinical scores were assessed: Oxford Knee Score (OKS), Hospital for Special Surgery Score (HSS), Lequesne Score (LEQU), and Tegner Activity Scale (TEG). Moreover, the SF-36 questionnaire was used to evaluate the patients’ subjective HRQL. Complications were defined as arthrofibrosis, thrombosis, pulmonary embolism, implant failure, and surgical-site infection as well as hematomas that required additional surgery.

The SF-36 questionnaire is used to determine HRQL and has been adjusted for age and gender. It allows the comparison of each patient to a person with similar demographic characteristics in the general population [25]. The norm-based evaluation requires transformation of the raw values. An individual result beneath 50 complies with a score beneath the specific national general population (GP), whereas results above 50 demonstrate a score above the specific national general population. In detail, the SF-36 questionnaire provides eight subscales for each element of the subjective HRQL, from which two summary scores can be formed. First, the physical component summary score (PCS) can be interpreted as the physical health, and, second, the mental summary score (MCS) can be interpreted as the mental health.

### Radiological evaluation

Anteroposterior and lateral radiographs of the knee as well as weight-bearing anteroposterior long-leg radiographs were taken at each follow-up, including the final follow-up after 6 years. The anatomical landmark-based digital planning software mediCAD® (Hectec, Landshut, Germany) was used to measure the mechanical tibiofemoral angle (mTFA). High test–retest reliability for mTFA using mediCAD has been previously proven [30]. The change in the mechanical limb alignment was expressed in absolute values and was calculated from the difference between the initial postoperative mTFA and the mTFA at the 6th-year follow-up.

### Surgical therapy

A landmark-based deformity analysis using mediCAD preceded the procedure. Biplanar osteotomy as described by Staubli et al. as well as Lobenhoffer and Agneskirchner was performed in each case using the TomoFix™ MHT plate fixator (DePuySynthes, Solothurn, Switzerland) [23, 35]. All patients received partial postoperative weight-bearing from 11 days to 6 weeks based on the individual protocol of the surgeon. Postoperative management and further details concerning the performed procedure have been described in previous publications [28, 29].

### Statistics and data analysis

The statistical analysis was performed with IBM SPSS® Version 24 (Armonk, New York, USA). A normal distribution could be assumed according to the central limit theorem due to the sample size of *n* = 85. Consequently, parametric tests were used. Univariate variance analyses were applied to compare HRQL and clinical scores between the groups. The comparisons of the means were preceded by Levene’s tests for equality of variances. Post hoc tests with alpha-adjusted Bonferroni corrections or post hoc tests according to Games-Howell were applied, depending on the results from the Levene’s test. Chi-square tests were used to compare the groups in terms of conversion to TKA and perioperative complications. For frequencies smaller than five, the Fisher-Yates-exact test was applied. Differences between the groups regarding changes in outcome variables over the follow-up period were analyzed by dependent *t* tests as well as univariate variance analysis. Post hoc tests were calculated as stated above. Changes in the mechanical limb alignments after 6 years were analyzed in regard to the BMI groupings. To compare the means between postoperative mTFA and mTFA at the 6th-year follow-up, dependent *t* tests were used. The statistical significance level was set at *p* ≤ 0.05 for all tests.

## Results

### Study cohort characteristics

**Fig. 1:**
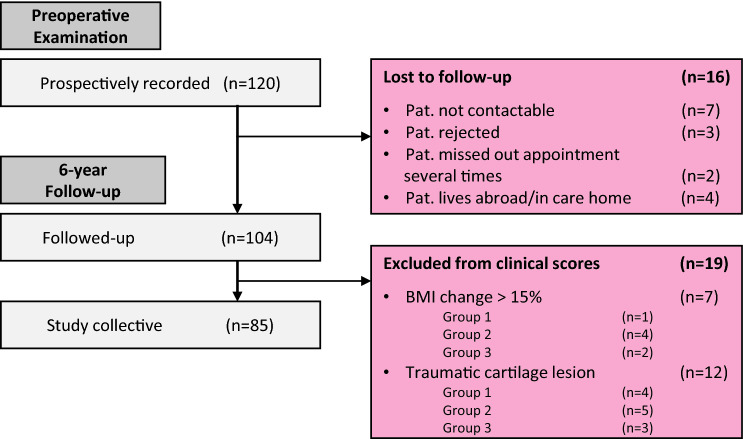
Flow chart showing the follow-up rate and the reasons for lost to follow-up and exclusion from the study

Of 120 patients, 85 had at least a 6-year follow-up (mean 6.7 years, range 6–11.8 years) after open-wedge HTO. Additionally, 19 patients were excluded for all further examinations according to the defined exclusion criteria (Fig. 1). The mean age was 54.6 years, while the average BMI was 28.6 ± 4.6 kg/m^2^. No significant differences were observed between the BMI groups for the recorded characteristics (age, affected side, sex, mTFA preOP, mTFA surgical correction, mTFA 6 years; *n.s.).* Further details of the study cohort characteristics and the study protocol are shown in Fig. 1 and Table 1.

**Table 1:** Detailed characteristics of the study collected from the BMI groupings

Characteristics	Total	Group 1 (BMI < 25 kg/m^2^)	Group 2 (BMI 25–30 kg/m^2^)	Group 3 (BMI > 30 kg/m^2^)
Age [years]	54.6 ± 6.4	54.3 ± 7.4	54.8 ± 6.0	54.6 ± 6.5
Affected side				
Left (%)	37 (44)	8 (44)	17 (46)	12 (40)
Right (%)	48 (56)	10 (56)	20 (54)	18 (60)
Sex				
Male (%)	60 (71)	11 (61)	29 (78)	22 (73)
Female (%)	25 (29)	7 (39)	8 (22)	8 (27)
mTFA^a^				
mTFA preOP [°]	− 4.9 ± 2.2	− 5.3 ± 2.6	− 4.9 ± 2.1	− 4.7 ± 2.1
mTFA surgical correction [°]	6.9 ± 3.2	7.5 ± 2.8	6.3 ± 2.9	7.3 ± 3.8
mTFA 6 years [°]	0.4 ± 2.6	1.0 ± 2.3	0.0 ± 2.6	0.7 ± 2.7

All metric values are arithmetic means ± SD

*mTFA* mechanical tibiofemoral angle, *mTFA surgical correction* the difference between the preoperative mTFA and the initial postoperative mTFA

^a^5 patients were not followed-up radiologically at the final follow-up

### BMI and HRQL

#### Mental aspect of HRQL

**Fig. 2:**
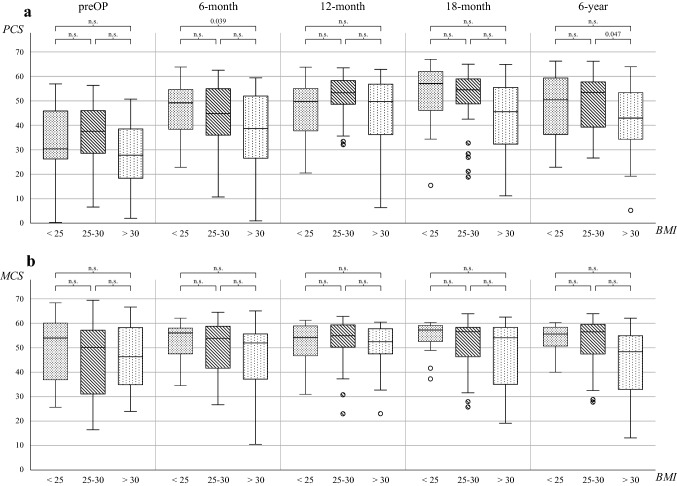
Total scores of PCS (**a**) and MCS (**b**) during the period under review for the BMI graduated groups. *PCS* physical component summary score, *MCS* mental component summary score, *preOP* preoperative recording, *n.s.* not significant

Patients in groups 2 and 3 showed lower preoperative values as compared to the referenced GP (Table 2). During the follow-up period, patients of all groups improved up to the 18th month follow-up in regard to the mental aspects but only group 1–2 patients were able to maintain high values over time (Fig. 2b, Appendix). After 6 years, a reduced mental condition was found for obese patients (group 3) as compared to the GP (Table 2).

**Table 2:** BMI groups and their respective deviation from the general population at two points in time

preOP—GP					6 year—GP			
Total		BMI < 25	BMI 25–30	BMI > 30	Total	BMI < 25	BMI 25–30	BMI > 30
MCS	− 3.2 ± 13.3^a^	− 0.1 ± 13.3	− 4.4 ± 13.8	− 3.7 ± 12.8	− 0.32 ± 12.3	1.7 ± 10.5	2.4 ± 10.1	− 5.1 ± 14.7
*p* value	0.025	n.s	n.s	n.s	n.s	n.s	n.s	n.s
PCS	− 17.6 ± 14.5^a^	− 18.6 ± 17.5^a^	− 14.2 ± 13.9^a^	− 21.1 ± 12.5^a^	− 3.2 ± 13.4^a^	− 2.2 ± 13.1	0.4 ± 11.5	− 8.6 ± 14.6^a^
*p *value	< 0.001	< 0.001	< 0.001	< 0.001	0.050	n.s	n.s	0.010

Preoperative values minus values of the general population (preOP–GP) and values of the 6th-year follow-up minus values of the general population (6 year–GP). PCS corresponds with the physical dimensions, while MCS corresponds with the mental dimensions of the subjective health-related quality of life

All values are arithmetic means ± SD

*GP* general population (adjusted for age and sex differences), *PCS* physical component summary score, *MCS* mental component summary score, *preOP* preoperative recording, *6 year* 6th-year Follow-up

^a^Paired differences are significant at the 0.05 level (2-sided)

#### Physical aspect of HRQL

In each group, a significant increase between the preoperative and 6th-year follow-up was found *(p* < *0.01 *for all groups)*.* The preoperative PCS differed significantly between groups 1 to 3 and the GP *(p* < *0.01 *for all groups*; *Table 2). At the 6th-year follow-up, the PCS of group 3 differed from that of the GP, while the PCS of groups 1–2 was similar to that of the GP (Table 2). Analyzing the change in PCS between the follow-ups revealed differences between the groups (Fig. 2a, Appendix): Up to 6 months, group 1 showed a higher improvement compared to groups 2–3. The highest values for groups 1 and 2 were found 18 months after surgery, whereas group 3 patients demonstrated their peak physical situation as soon as at the 12th-month follow-up. After reaching the peak value, all groups decreased in physical aspects until the 6th-year follow-up (Fig. 2a, Appendix). In total, patients of group 3 showed the best improvement from before the procedure to 6 years after the procedure, but showed inferior results compared to the highest peak values of *groups 1 and 2 (preOP to 6 years:* + *11.9* ± *17.2 *for group* 1,* + *9.9* ± *14.1 *for group 2 and + *13.7* ± *14.7 *for group 3). Moreover, a significant benefit was found in all groups after 6 years as compared to preoperatively *(p* < *0.01 for all groups).*

#### BMI and clinical outcome

**Fig. 3:**
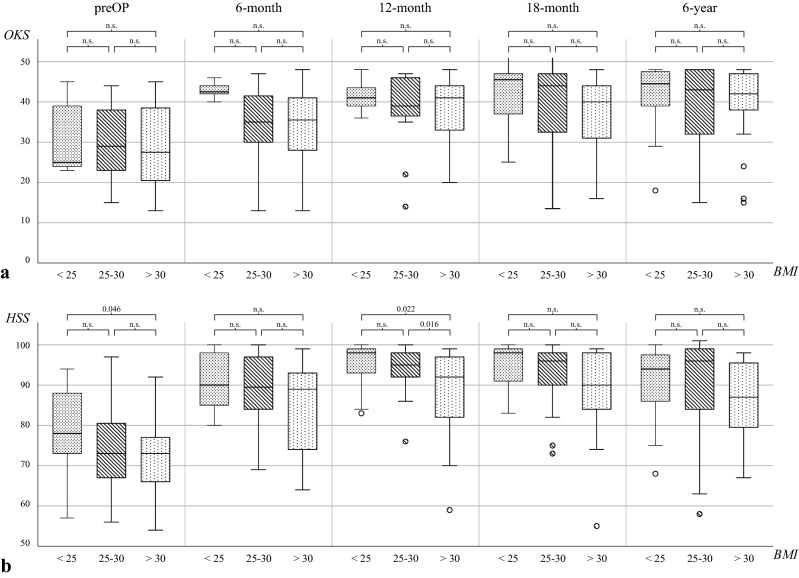
Total scores of OKS (**a**) and HSS (**b**) during the period under review for the BMI graduated groups. *OKS* Oxford knee score, *HSS* hospital for special surgery score, *preOP* preoperative recording, *n.s.* not significant

The whole cohort as well as each BMI group showed a significant increase in all recorded scores from before the procedure to the 6th-year follow-up *(p* < *0.01; *Fig. 3a and b, Table 3). At the last follow-up, no significant difference was found between groups 1–3 in all clinical scores but a tendency toward inferior results in overweight patients (group 3) can be observed (*n.s.*). However, overweight patients have already started from a lower level in all clinical scores (Fig. 3a and b, Table 3).

**Table 3:** Total scores of the LEQU and TEG for the BMI groups

Score	Follow-up	Total	BMI < 25	BMI 25–30	BMI > 30	*p* value (significance between groups)		
						BMI < 25 vs. BMI 25–30	BMI < 25 vs. BMI > 30	BMI 25–30 vs. BMI > 30
LEQU								
	preOP	8.7 ± 4.4	8.5 ± 4.0	8.0 ± 5.0	9.8 ± 3.6	n.s	n.s	n.s
	6-month	4.8 ± 4.2	3.6 ± 3.8	3.9 ± 4.2	6.7 ± 3.9	n.s	0.031	0.015
	12-month	3.0 ± 3.6	2.3 ± 3.1	2.3 ± 2.5	4.3 ± 4.7	n.s	n.s	n.s
	18-month	3.1 ± 4.0	1.9 ± 3.0	2.2 ± 3.5	4.7 ± 4.7	n.s	n.s	0.029
	6-year	3.8 ± 3.7	3.6 ± 3.8	3.3 ± 4.1	4.5 ± 3.2	n.s	n.s	n.s
TEG								
	preOP	2.9 ± 1.1	3.0 ± 1.9	3.0 ± 1.0	2.8 ± 0.7	n.s	n.s	n.s
	6-month	3.4 ± 1.1	3.6 ± 1.0	3.6 ± 1.2	3.1 ± 0.9	n.s	n.s	n.s
	12-month	3.5 ± 0.9	3.6 ± 0.8	3.7 ± 1.0	3.2 ± 0.6	n.s	n.s	0.050
	18-month	3.6 ± 1.0	4.1 ± 0.9	3.8 ± 1.0	3.2 ± 1.0	n.s	0.024	n.s
	6-year	3.8 ± 1.2	3.9 ± 1.1	3.9 ± 1.5	3.5 ± 1.0	n.s	n.s	n.s

All values are arithmetic means ± SD

Significance level *p* ≤ 0.05 (2-sided)

*LEQU* lequesne score, *TEG* tegner activity score, *preOP* preoperative recording, *n.s. *not significant

#### Conversion to TKA and general complications

Out of 85 patients, 9 (10.5%) converted TKA 50.1 ± 25.0 months following HTO surgery. These included 2 from the 18 patients in *group 1* (11.1%; 57.0 months after surgery), 3 of the 37 in group 2 (8.1%; 49.7 months after surgery), and 4 of the 30 patients in *group 3* (13.3%; 48.3 months after surgery). However, there was no significance regarding frequency of conversion to TKA between groups 1 to 3* (n.s.).* Patients with conversion to TKA (29.0 ± 4.6 kg/m^2^) had a slightly higher, but not significantly different BMI compared to patients without conversion to TKA (28.5 ± 4.6 kg/m^2^; *n.s.).* In total, five complications occurred (one hematoma and four infections on the surgical site). The hematoma required a second intervention, but subsequently healed without consequence. The infections were subcutaneous infections that did not require further surgery and also healed without consequence. No significant difference between BMI groups was found (BMI < 25: *n* = 1, BMI 25–30: *n* = 2, BMI > 30: *n* = 2; *n.s.)*.

#### BMI and loss of correction

A mean correction difference of 6.9° ± 3.2° (mTFA) compared to the preoperative value was documented six weeks after the open-wedge HTO (Table 1). The correction difference throughout the groups was relatively constant and showed no significant differences *(n.s.).* The change in mechanical limb alignment showed a significant difference between the initial postoperative values and the 6th-year follow-up in each group *(p* = *0.033 *for group 1,* p* < *0.001 *for group 2,* p* = *0.012 *for group 3). Patients in *group 3* showed significantly higher changes in mTFA than the patients with normal weights during the follow-up period over 6 years (mTFA: 0.9° ± 1.5° for group 1, 1.8° ± 1.5° for group 2, 2.2° ± 3.7° for group 3; *p* = *0.032).*

## Discussion

The most important finding was that overweight patients achieved inferior clinical and functional mid-term results after open-wedge HTO. However, obese patients demonstrated an improvement after open-wedge HTO to the same extent as patients with normal weights. In overweight patients, mental and clinical scores did not reach the peak values of the normal weight population. A higher frequency in conversion to TKA as well as a significantly higher loss of correction over time was demonstrated *(p* < *0.05)*. Considering the inferior preoperative clinical scores and HRQL of obese patients, the present study revealed an improvement after open-wedge HTO to the same extent as the patients with normal weights. Consequently, overweight patients can benefit from open-wedge HTO.

In accordance with our results, several studies have also reported inferior clinical and functional short- to mid-term results for obese patients following open-wedge HTO [2, 6, 9, 34]. Nevertheless, no previous study has described the effect of being overweight on clinical, radiological, and psychological parameters based on a large and prospectively assessed clinical trial 6 years after open-wedge HTO. The analysis of BMI as a risk factor for poor clinical outcomes has to take into account that, in general, obese patients show a reduced quality of life, particularly regarding the physical dimensions [8]. This can explain the remaining differences after surgery between obese patients and the GP in physical dimensions of the SF-36. However, significant improvements in clinical scoring for obese patients were also demonstrated *(p* < *0.05)*. These pre- to postoperative results were comparable or even superior to patients with normal weights. Therefore, HTO is a suitable treatment option for overweight patients; however, a detailed patients’ education prior to surgery is mandatory to set realistic expectations for the patients. Our results were in accordance with the 3-year results described by Floerkemeier et al. [6]. Their study revealed favorable results with similar complication rates even in smokers and obese patients. Quality of life, conversion to TKA, and loss of correction over time were not assessed. A limitation in the study protocols in both our and their studies is that no morbidly obese patients (morbid obesity corresponds with obesity class II and higher, with a BMI ≥ 35 kg/m^2^ as well as experiencing obesity-related health conditions [22]) were evaluated in detail. In our study, morbidly obese patients were also included in the group BMI > 30 kg/m^2^ without a separate group for patients with BMI ≥ 35 kg/m^2^. A potential tendency for clinical results to be inferior as well as higher complication rates has to be considered for this group for this reason.

To differentiate between the influence of BMI on the surgical outcome and the inferior function of patients with high BMI in general, a prospective study design that compares the mid-term surgical outcomes with the initial preoperative baseline is needed. Our prospective study was focused on the impact of BMI on outcome measures following open-wedge HTO to treat unicompartmental varus osteoarthritis of the knee. The difference between the preoperative score and that of the 6th-year follow-up is an important measure to evaluate treatment success in this specific cohort. In the patient group with a BMI > 30 kg/m^2^, OKS, HSS, LEQU, and TEG improved similarly to patients with lower BMI. In PCS, the group with a BMI > 30 kg/m^2^ achieved the greatest improvement from all groups until the 6th-year follow-up. Moreover, the evaluation of subjective outcomes and mental health in patients with degenerative joint diseases is important [16, 27]. In our study, the MCS, as a score for mental health, was compared to the scores of the GP. The groups with a BMI < 25 kg/m^2^ and between 25 and 30 kg/m^2^ were already similar to the GP preoperatively, such that significant improvement was not expected. Obese patients had constant values over the observation period, but they were lower compared to the GP. This could be explained by a longer postoperative duration of increased pain levels and delayed clinical and functional improvements. This can result in dissatisfaction and emphasizes the importance of realistic and individualized patient education to prevent dissatisfaction caused by false expectations [11, 13, 16].

The influence of patients’ BMI on perioperative and postoperative complications following HTO or similar procedures, has been discussed controversially in previous literature [2, 6, 24]. The results of our study showed no significant relationship between BMI and the complication rate *(n.s.).* However, the primary aim of this study was not to assess the specific complications in obese patients, and only major complications were recorded.

Since being overweight has been shown to lead to faster progression of osteoarthritis [3, 5], outcomes of obese patients following HTO surgery in the long-term may be affected. Thus, earlier and more frequent conversion to TKA should be observed in the long-term. Previous studies are limited to those with a retrospective study design, small sample sizes, short follow-up period, or low follow-up rate [2, 6, 18], and the numbers of patients with a conversion to TKA are often too small to calculate risk factors reliably [24]. Therefore, more prospective studies are needed. In our study, only nine patients underwent TKA over the follow-up period. Consequently, conclusions regarding the relationship between BMI and conversion to TKA cannot be drawn.

The influence of BMI on postoperative changes in the mechanical limb alignment was illustrated by the study’s results. This underlines the resounding suggestion of previous publications to use iliac crest bone grafts in high-risk patients [14, 24]. For example, Siboni et al. reported obesity as a risk factor for the non-union of open-wedge HTO with large correction greater than 10° without filling the osteotomy gap [32]. They recommended grafting of a systematic bone or bone substitute in overweight patients in cases with a > 10° mechanical correction. Further research is needed to analyze the impact of mechanical limb alignment changes in clinical and functional outcomes and earlier conversion to TKA.

Besides analyzing the clinical scores, complication rates, or radiographic outcomes in obese patients, concurrent therapy options for obese patients with unicompartmental varus gonarthritis have to be considered. The gradual approach to treat unicompartmental varus gonarthritis includes medial open-wedge HTO as well as unicompartmental knee arthroplasty as the possible preliminary stages on the way to TKA (total knee arthroplasty) [13]. As demonstrated by our results, obese patients with unicompartmental varus gonarthritis can benefit from open-wedge HTO. There may be differences in the outcome to the normal weight population but acceptable treatment alternatives for obese patients are limited, given that obese patients treated with unicompartmental knee arthroplasty have a significantly inferior surgical outcome and a higher risk of developing an adverse event [26]. Moreover, inferior outcomes in patients with obesity following primary TKA have been shown by many previous studies [17, 19, 33].

This study has some limitations. First, it was a post hoc analysis of prospectively collected data. The initial sample size calculation was based on the comparison of two different postoperative treatment protocols. Non-significant differences between the three BMI groups could be a result of low power and a type II error. Moreover, the study had no control group with an alternative treatment, such as unicompartmental knee arthroplasty. Furthermore, all patients were randomized to two postoperative treatment protocols, which may result in biased results, as randomization was not stratified to BMI. Early weight-bearing may cause early correction loss, especially in overweight patients. However, the different post-treatment protocols had no influence on surgical accuracy (absolute mTFA deviation of the postoperative achieved correction from the intended correction), and also not to the loss of correction in the entire observation period. Additionally, a significant difference in clinical scores between the treatment groups was found 6 months after surgery. After twelve months, no significant difference was observed [28]. Besides our outcome parameters, further parameters should be considered in obese patients. For example, the time to union and non-union rates was not reported in our study, but should be critically assessed in these patients [32]. Moreover, we did not stratify between the BMI groups in regard to the preoperative degree of osteoarthritis and area of cartilage defects. However, we excluded patients with bone-related posttraumatic osteoarthritis.

Despite these limitations, the study is of particular clinical relevance because overweight patients achieve the same clinical and functional improvements as to that in patients with normal weights within the first 6 years. An open-wedge HTO should therefore not be excluded for overweight patients in general. The overall clinically and functionally lowered level in overweight patients must be communicated to them through individualized patient education. The development of a treatment concept along with a realistic expectation horizon together with the patient is mandatory.

## Conclusion

Overweight patients have lower clinical and functional preoperative and mid-term results after open-wedge HTO compared to normal weight patients. Mental as well as clinical scorings did not reach the postoperative peak values of the normal weighted population. A higher frequency in conversion to TKA may be possible, while a higher loss of correction over time is to be expected. Nevertheless, in terms of clinical outcomes and HRQL, overweight patients can benefit from open-wedge HTO to the same extent as patients with normal weights and demonstrate similar complication rates.

## Appendix

See Table

**Table 4:** Total scores of the PCS, MCS, OKS and HSS for the BMI groups

Score	Follow-up	Total	BMI < 25	BMI 25–30	BMI > 30	*p* value (significance between groups)		
						BMI < 25 vs. BMI 25–30	BMI < 25 vs. BMI > 30	BMI 25–30 vs. BMI > 30
PCS								
	preOP	32.4 ± 14.5	31.4 ± 17.5	35.8 ± 13.9	28.9 ± 12.5	n.s	n.s	n.s
	6-month	41.7 ± 14.6	46.9 ± 10.7	43.4 ± 13.6	36.2 ± 16.4	n.s	0.039	n.s
	12-month	47.7 ± 13.3	46.7 ± 12.1	50.3 ± 12.0	44.9 ± 15.1	n.s	n.s	n.s
	18-month	48.5 ± 13.6	52.1 ± 13.9	50.6 ± 13.0	43.9 ± 13.5	n.s	n.s	n.s
	6-year	46.8 ± 13.4	47.8 ± 13.1	50.4 ± 11.5	41.4 ± 14.6	n.s	n.s	0.047
MCS								
	preOP	46.8 ± 13.3	49.9 ± 13.3	45.6 ± 13.8	46.3 ± 12.8	n.s	n.s	n.s
	6-month	49.4 ± 12.1	52.7 ± 8.0	50.1 ± 12.0	46.4 ± 14.0	n.s	n.s	n.s
	12-month	51.2 ± 11.2	52.0 ± 8.4	52.1 ± 11.4	49.5 ± 12.4	n.s	n.s	n.s
	18-month	50.7 ± 11.0	54.6 ± 6.8	51.6 ± 9.9	47.4 ± 13.3	n.s	n.s	n.s
	6-year	49.7 ± 12.3	51.7 ± 10.5	52.4 ± 10.1	44.9 ± 14.7	n.s	n.s	n.s
OKS								
	preOP	29.8 ± 9.4	31.3 ± 9.4	30.2 ± 8.9	28.7 ± 10.4	n.s	n.s	n.s
	6-month	35.7 ± 8.8	42.8 ± 2.0	35.2 ± 8.3	33.3 ± 9.9	n.s	n.s	n.s
	12-month	38.8 ± 8.2	41.4 ± 4.0	39.1 ± 8.8	37.5 ± 8.9	n.s	n.s	n.s
	18-month	39.7 ± 10.3	43.3 ± 10.4	40.5 ± 10.5	37.5 ± 10.0	n.s	n.s	n.s
	6-year	39.7 ± 9.1	41.5 ± 8.2	39.0 ± 9.4	39.5 ± 9.3	n.s	n.s	n.s
HSS								
	preOP	74.0 ± 10.2	78.7 ± 10.1	73.6 ± 10.7	71.5 ± 9.0	n.s	0.046	n.s
	6-month	88.1 ± 9.2	90.8 ± 6.9	89.5 ± 7.9	84.6 ± 11.0	n.s	n.s	n.s
	12-month	91.6 ± 8.6	94.4 ± 5.9	93.4 ± 7.2	87.6 ± 10.4	n.s	0.022	0.016
	18-month	91.2 ± 10.7	94.8 ± 5.7	92.4 ± 9.6	87.6 ± 13.2	n.s	n.s	n.s
	6-year	88.5 ± 11.8	90.6 ± 9.2	90.0 ± 12.4	85.2 ± 12.2	n.s	n.s	n.s

All values are arithmetic means ± SD

Significance level *p* ≤ 0.05 (2-sided)

*PCS* physical component summary score, *MCS* mental component summary score, *OKS* Oxford knee score, *HSS* hospital for special surgery score, *preOP* preoperative recording, *n.s. *not significant

4.

## Abbreviations

BMI: Body mass index
GC: General complications
GP: General population
HRQL: Health-related quality of life
HSS: Hospital for Special Surgery Score
HTO: High tibial osteotomy
LEQU: Lequesne Score
MCS: Mental component summary score
mTFA: Mechanical tibiofemoral angle
OKS: Oxford Knee Score
PCS: Physical component summary score
TKA: Total knee arthroplasty
